# Physical, cognitive, social and mental health in near-centenarians and centenarians living in New York City: findings from the Fordham Centenarian Study

**DOI:** 10.1186/s12877-015-0167-0

**Published:** 2016-01-05

**Authors:** Daniela S. Jopp, Min-Kyung S. Park, Jonathan Lehrfeld, Michelle E. Paggi

**Affiliations:** University of Lausanne, Lausanne, Switzerland; Kingsborough Community College, Brooklyn, NY USA; Fordham University, Bronx, NY 10458 USA; Weill Cornell Medical College, New York, NY 10065 USA

**Keywords:** Centenarians, Health and well-being, Cognition, Social resources, Depression, Community and public health

## Abstract

**Background:**

Despite their strong increase, the population of the very old, including near-centenarians and centenarians, represent an unstudied and underserved population. Available studies mostly concentrate on predictors of exceptional longevity, but rarely extend their focus to other areas of functioning. Also, little is known about what contributes to experiencing a quality life in very old age. The present population-based study aims at providing a comprehensive picture of key domain of functioning, including physical, cognitive, social and mental function in very old individuals and to determine predictors of mental health indicators.

**Methods:**

A total of 119 individuals aged 95 to 107 living in private dwellings and residential care facilities were recruited based on the New York City Voters Registry. Participants answered questions regarding their health and activities of daily living. Their cognitive functioning was determined using the Mini-Mental State Examination and the Global Deterioration Scale. Social resources were measured with number of children and the Lubben Scale. Mental health was assessed with the Geriatric Depression Scale and the Satisfaction with Life Scale.

**Results:**

An unexpectedly large proportion of the sample lived in the community. On average, cognitive functioning was high. Although five diseases were reported on average, participants reported good health. Functional status was reduced. Most participants had at least one person for communication/social support. On average, depression was below cut-off, and most participants reported high life satisfaction. Regression analyses indicated that individual differences in depression were associated with subjective health, IADL and relatives support. For life satisfaction, subjective health, ADL and number of children were most important. Demographic characteristics, number of illnesses or cognitive status were not significant.

**Conclusions:**

Despite reduced levels of physical functioning and social resources, very old participants were in good mental health suggesting high resilience and ability to adapt to age-associated challenges. That a large proportion of them lived in the community further highlights their desire for leading an autonomous life, which may have been facilitated by New York service culture. More research is necessary to provide guidance for the development of well-suited services for this very old population.

## Background

In most developed countries, increasing longevity has led the very old to become the fastest growing segment of the population. For example, in the US, the number of centenarians increased by 65.8 % between 1980 and 2010, resulting in a total of 53,364 individuals aged 100 or older [1]. This trend is expected to accelerate, as every other child born after the year 2000 is likely to reach her/his 100^th^ birthday [2]. Despite this global demographic development, very old individuals including centenarians remain an understudied and underserved population. The few centenarian studies available mostly concentrated on physical health, including functional and cognitive limitations, and potential predictors of longevity [3–9]. Little is known about other areas of functioning, such as mental health, and to what extent physical health, cognition or social resources play a role [10]. A broad and representative knowledge base is urgently needed to better understand what it means to live to age 100 and how this experience can potentially be influenced. The main goal of the present study was to create a comprehensive picture of very old individuals (95 years and older) by describing central areas of functioning (health, cognition, social functioning, and mental health) and by investigating which health and psychosocial characteristics were most important for explaining individual differences in mental health indicators (i.e., depression and life satisfaction).

Although reaching age 100 is mostly seen as a considerable achievement, whether centenarians represent a model of successful aging is under scientific debate. On the one hand, Evert and colleagues [11] reported that some centenarians experienced delayed onset of age-related illnesses (delayers) while others did not succumb to any age-related illnesses (escapers). In addition, Anderson and colleagues [12] found that younger age groups and centenarians would spend more years living with illnesses than supercentenarians (i.e., 105 years and older). On the other hand, centenarians’ actual physical health is often severely compromised. For example, Andersen-Ranberg and colleagues [13] identified only one physically healthy centenarian in their sample of over 200 Danish participants. Thus, besides living longer and healthier lives, many are confronted with health conditions in very advanced age.

Centenarians’ cognitive functioning is also a key area of interest. As the prevalence of dementia increases dramatically between ages 65 and 85, one would assume that most centenarians have moderate to severe cognitive limitations. Research evidence shows that this is not the case: various studies have documented that there are centenarians who are free of cognitive impairment [6], though prevalence rates of cognitive impairment vary substantially across studies, ranging between 34 % [10] to 80 % [14, 15]. The percentage of centenarians with severe cognitive impairment has been reported to be between 10 % [10] and 40 % [14, 16].

Research on centenarians to date offers little information on other factors that may contribute to longevity, such as social resources. Social partners are essential during all phases of life, but their importance for maintaining autonomy and well-being may become more salient in advanced age due to an increase of age-related health conditions. Unfortunately, due to their exceptional age, widowhood and loss of a child are very likely in centenarians [17]. The Georgia Centenarian Study also found evidence of reduced social partners (e.g., people to visit or talk on the phone) in centenarians relative to octogenarians [18]. At the same time, both the Heidelberg Centenarian Study and the Georgia Centenarian study showed that social resources were predictive of self-rated mental and physical health [19] and happiness [20].

Research on mental health in the very old is also lacking as only few studies have addressed whether centenarians experience their lives as satisfying and worth living despite age-related health restrictions and social loss. Research on depressive symptoms is also limited and inconsistent, with prevalence rates ranging between 13 % in an Italian sample [21] to 25 % in the Georgia Centenarian Study [22]. Qualitative insights on centenarians’ well-being and related factors are for example available from centenarian living in Hongkong [23]. In the Heidelberg Centenarian Study, centenarians reported high levels of well-being and were found to be as happy as middle-aged adults, despite health limitations. In fact, it turned out that health did not predict individual differences in happiness [20]. At the same time, various centenarians in the sample reported depressive symptoms [24].

In order to gain a comprehensive portray of central areas of functioning in very advanced age, the first aim of the present study was to investigate levels of health, cognitive, social and mental function in a US sample of near-centenarians and centenarians. The second aim was to examine the extent to which health, cognitive, social and mental functioning relate to each other. The third aim was to identify which health, cognitive and social variables best explain interindividual differences in mental health indicators (i.e., depression and life satisfaction).

## Methods

### Sample

A total of 119 individuals aged 95 to 107 (M_Age_ = 99.25) participated in the Fordham Centenarian Study (78.2 % females) (Table 1). Ethnicity distribution was 79.8 % White, 19.3 % African American and .8 % “other.” Four individuals (3.4 %) self-identified as Hispanic. We recruited our sample from a list of individuals 95 years and older who had registered to vote in New York City.

**Table 1:** Demographic characteristics of the sample

	N (%)
Study participants	119 (100)
Near-centenarians (95–99 years)	57 (48)
Centenarians (100–107 years)	62 (52)
Gender	
Females	93 (78)
Males	26 (22)
Ethnic background	
Caucasian	95 (80)
African American	23 (19)
Other	1 (1)
Residence type	
Community-dwelling	88 (74)
Institutionalized	31 (26)
Marital status	
Never married	11 (9)
Married	8 (7)
Divorced	9 (8)
Separated	2 (2)
Widowed	89 (75)
Education	
Kindergarten or primary school	3 (3)
Middle school	23 (20)
9^th^ to 12^th^ Grade (no Diploma)	13 (11)
High school diploma	21 (18)
Some college vocational	17 (15)
Bachelor’s	21 (18)
Graduate school or higher	19 (16)
U.S. or foreign-born	
U.S.-born	84 (71)
Foreign-born	35 (29)

Specifically, we sent out 1,032 recruitment letters to individuals in the three most diverse boroughs: Manhattan, Bronx, and Brooklyn. We called one week later and reached 492 (48 %) potential participants or their families. (The main reason for the fact that we were only able to verify the existence of about half of those invited to the study was that the Voter’s Registry seems not well-updated, and individuals who had deceased long ago were still included). In order to be eligible for the study, participants did not need to be fully cognitively intact but able to reliably respond to questions about themselves, as we were interested in the very olds’ experience of their very advanced age, well-being and depressive symptoms as well as other psychological constructs. Out of 492 target persons (or their families) reached by telephone, 159 (32 %) were ineligible: the largest group had deceased before telephone contact could be established (60 %), had severe cognitive impairment according to family members (21 %) or very poor physical health (19 %). Of the 320 eligible individuals (i.e., not necessarily cognitively fully intact but able to provide reliable information about themselves), 204 (64 %) refused and 116 (36 %) agreed to participate. Refusals were mostly due to no interest in the study (67 %), no time (12 %) and other reasons (21 %). Thirteen individuals who agreed to participate were not interviewed (2 deceased, 2 experienced illness downturns, 3 changed their mind, 1 relative intervened, 5 could no longer be reached). This recruitment approach resulted in 103 interviews, of which 95 were included in the study (7 had severe cognitive impairment not obvious during phone contact, 1 withdrew after family intervention). As the just described main recruitment approach made it slightly more difficult to reach individuals in nursing homes, we recruited an additional 23 participants via five collaborating health care providers. Finally, one additional centenarian was recruited by word of mouth, resulting in a total 119 study participants. Interviews were divided into two sessions of 1.5 h each to minimize fatigue and were conducted at the participant’s residence after giving informed consent in writing. Study procedures were approved by three Institutional Review Boards (Fordham University, Jewish Home Lifecare, Hebrew Home for the Aged).

### Measurements

Interviews covered four main areas of functioning, including (a) physical functioning, (b) cognitive functioning, (c) social resources, and (d) mental health.

- Physical functioning: Physical functioning was indicated by number of diagnoses, subjective health, and functional status. Participants responded “yes or no” to a checklist of common age-related illnesses. Health conditions included: high blood pressure, heart condition, diabetes, chronic lung disease, ulcers or other serious stomach issues, cirrhosis or other liver problems, kidney condition, frequent urinary infections, incontinence, prostate problems, problems with vision or hearing, arthritis, osteoporosis, stroke, cancer, pneumonia, falls, and other. Conditions mentioned as “other” were later coded. Subjective health was measure d with an item asking participants to evaluate their current health status (1 = poor; 5 = excellent). Functional status was measured with the Older Americans Resources and Services (OARS) Multidimensional Functional Assessment Questionnaire [25] in which participants were asked how much difficulty they had performing seven personal activities of daily living (PADLs) and seven instrumental activities of daily living (IADLs) using a 3-point rating scale (0 = can’t do without help, to 2 = no difficulty; 0–14 each).
- Cognitive functioning: To assess cognitive functioning, we used the following subscales from the Mini-Mental State Examination (MMSE) [26]: Orientation (range: 0–10 points), Registration (range: 0–3 points), Attention (0–5 points), and Recall (0–3 points), resulting in a maximum total of 21 points. We followed the recommendations by Holtsberg et al. [27], who proposed using items that were unlikely to be biased by the poor sensory functioning highly prevalent in centenarians. This selection of MMSE items has been used in prior centenarian studies [16]. As a second cognitive indicator, we used the Global Deterioration Scale (GlobDetScale) [28], which is an observer’s rating of cognitive status (1 = no memory deficit evident from interview, to 7 = very severe cognitive decline).
- Social resources: Number of living children was used as an indicator of social resources. Social contact and support was assessed with the 6-item Social Network Scale [29]. Items asked for the number of relatives and number of friends to whom one talks to at least once a month, with whom one feels at ease to talk with about private matters (confidants), and to whom one feels close enough to ask for help (SOS contacts; 0 = none, to 5 = nine or more).
- Mental health: We used the 15-item version of the Geriatric Depression Scale (GDS) [30] to assess depressive symptoms. Items were answered using 1 = yes, and 0 = no and were summed; higher values indicated higher frequency of depressive symptoms (range: 0–15). Life satisfaction was measured with a modified version of the 5-item Satisfaction with Life Scale [31]. As centenarians with poor cognition had difficulty understanding items formulated as statements (e.g., In most ways, my life is close to my ideal), we reformulated those into questions (e.g., In most ways, is your life close to your ideal?). To further reduce cognitive load, we also limited the answering format to 5 options (0 = not at all, to 4 = very much). Higher mean scores represent greater subjective well-being.

### Statistical analyses

Complementing descriptive statistics, we tested gender differences with t-tests (mean levels) and Pearson’s chi-square tests (frequencies). Relationships between variables were examined with Pearson (continuous variables) and point-biserial (dichotomous variables) correlations. The extent to which specific variables were related to individual differences in mental health was examined with multiple regression analyses with either depression or life satisfaction as dependent variable. As the sample size did not permit including all variables simultaneously, we first conducted domain-specific models (i.e., health variables as predictors only) and then used all predictors from those domain-specific models with at least marginal predictive value (*p* ≤ .20) in a combined model. This model was further revised by including only predictors with *p* ≤ .10 to ensure an acceptable cases-to-predictor ratio.

## Results

### Demographic characteristics and living situation

The majority of the sample was community-dwelling (*n* = 88, 74 %) and widowed (*n* = 89, 75 %; Table 1). Participants were relatively well-educated, with one third (*n* = 40, 34 %) having a bachelor’s or higher degree and another third having a high school diploma or some college (*n* = 38, 32 %). Another third had middle school education or completed some high school (*n* = 36, 30 %). Very few (*n* = 3, 3 %) had received only primary school education. Most participants had been born in the U.S. (*n* = 84, 71 %).

**Table 2:** Central study variables: Number of participants providing information, Observed range, Mean level, Standard deviation

Key study variables	N	Min	Max	Mean (SD)
Number of illnesses^a^	116	0	11	4.85 (2.32)
Subjective health^b^	119	1	5	2.97 (1.12)
PADL^c^	108	0	14	10.41 (3.67)
IADL^d^	102	0	14	8.89 (4.05)
MMSE^e^	119	5	21	16.45 (4.04)
GlobDetScale^f^	100	1	5	1.44 (.91)
Number of living children^g^	119	0	6	1.36 (1.31)
Family support score	95	0	15	7.11 (3.44)
Friends support score	95	0	15	5.25 (4.41)
GDS^h^	107	1	14	4.10 (3.41)
Life satisfaction^i^	112	0	4	2.07 (1.14)

^a^Health conditions included: high blood pressure, heart condition, diabetes, chronic lung disease, ulcers or other serious stomach issues, cirrhosis or other liver problems, kidney condition, frequent urinary infections, incontinence, prostate problems, problems with vision or hearing, arthritis, osteoporosis, stroke, cancer, pneumonia, falls, and other. Within the “other” category, 20 additional conditions were mentioned. The resulting theoretical range was 0–36. ^b^subjective health theoretical range: 1–5. ^c^ *PADL* Personal Activities of Daily Living (range: 0–14). ^d^ *IADL* Instrumental Activities of Daily Living (range: 0–14). ^e^ *MMSE* Mini-Mental State Examination (shortened, range: 0–21). ^f^ *GlobDetScale* Global Deterioration Scales (range: 1–7). ^g^Number of living children: 6 indicates 6 or more. Due to an outlier with more than 20 children (biological and adopted), this variable had to be curtailed. ^h^ *GDS* Geriatric depression scale (range: 0–15). ^i^Life satisfaction range 0–4. *SD* Standard Deviation

### Physical functioning

Participants reported an average of 4.85 ± 2.32 illnesses (Table 2), indicating that many individuals dealt with multimorbidity. Poor health conditions were further highlighted by the fact that one fifth of the sample had seven to eleven health issues. Considering those with fewer health problems, there were only two individuals who did not indicate any health issues. About one fourth of the sample reported only up to three illnesses; still being faced with three chronic health issues concurrently is likely to go along with substantial burden. In contrast to the high illnesses count, participants’ subjective health was relatively high at 2.97 ± 1.12, with 67 % reporting good to excellent health. Functional health was relatively high, with an average of 10.41 ± 3.67 out of 14 from the PADL scale. Twenty-eight percent of the sample had the highest score of 14, and 21 % indicated difficulty with only 1 activity, and 19 % of the sample reported difficulty with only 2 activities. IADLs were slightly more impaired, with an average score of 8.89 ± 4.05; however, 17 % of the sample reported no difficulty. For specific activities, the basic personal activities most often reported as difficult were taking a bath (55 %), getting dressed (52 %) and moving in and out of bed (48 %). Difficulty on the other basic activities was reported by 20 % to 30 % of the sample. Most of the instrumental activities were considered difficult by at least 60 % of the participants. The only exception was talking on the phone, with  28 % of the sample indicating difficulty. The instrumental activities causing difficulty most often were light housework (77 %), shopping (76 %), preparing meals (65 %) and getting around/traveling (64 %).

### Cognitive functioning

The mean score of the shortened MMSE was 16.45 ± 4.04 (out of 21; Table 2). The mean Global Deterioration Scale score was 1.44 ± .91 (out of 7), also indicating few cognitive restrictions. Ninety-three individuals (93 %) had no or little cognitive limitations (scores of 1 to 3), and seven individuals (7 %) had moderate limitations (scores of 4 and 5).

### Social resources

The mean number of living children was 1.36 ± 1.31. On average, participants reported having three relatives to talk to at least once a month (2.97 ± 1.30), two relatives as confidants (2.03 ± 1.36), and two relatives as SOS contacts (2.18 ± 1.46; Table 2). Relative to family contacts, the number of friends available to talk at least once a month was smaller (2.32 ± 1.77), as was the number of friends being confidants (1.45 ± 1.54) and SOS contacts (1.53 ± 1.70). To compare the amount of social support received by near-centenarians and centenarians with younger individuals in prior studies, we combined the variables into a family, friends and total support sum score [29, 32]. On average, the total support score was 12.24 ± 6.36, the family support score was 7.11 ± 3.44 and the friends support score was 5.25 ± 4.41. Using 11 or less as cut-off for the total score and 5 or less for the family and friends score [29, 32], we found that, when considering their total network, half of the sample (51 %) was at risk for social isolation. Considering family support, the percentage at risk was smaller (34 %), but for friendship support, the risk was double (58 %).

### Mental health

Mental health was relatively high in this sample (Table 2). Mean depression score was 4.10 ± 3.41, and 72 % participants had few or no depressive symptoms (i.e., GDS scores 0–4). Over 80 % of the sample did not meet the criteria for clinical depression (GDS scores 8 and higher). Mean life satisfaction was 2.07 ± 1.14, indicating moderate life satisfaction. About 66 % reported moderate to very high life satisfaction. About 25 % were “a little” satisfied and only 9 % were not satisfied with their lives.

### Gender differences

There were no gender differences, except for marital status, *χ*^2^ (4, *N* = 119) = 8.99, *p* < .05, φ = .30: More males than females were still married.

### Predictors of mental health

Subsequently, we examined which variables predicted mental health. Table 3 includes correlations and Table 4 summarizes regression analyses. The domain-specific models indicate that demographic and cognitive variables had little predictive value and the overall models were not significant. The health variables models explained substantially more variance. Specifically, subjective health was a significant predictor of both depression and life satisfaction, and IADL was significant for depression and PADL for life satisfaction. The social variables model was significant only for depression, with a significant effect for Friends Support and a marginal effect for Relatives Support, but explained less variance than the health model.

**Table 3:** Correlations among central study variables

	1	2	3	4	5	6	7	8	9	10	11	12	13	14	15	16	17
Background variables																	
1. Age	––																
2. Gender	0.15***	––															
3. Residence (1 = priv, 2 = inst)	−0.05	0.08	––														
4. Widowed (1 = yes, 0 = no)	0.06	0.07	−0.05	––													
5. Education	−0.19*	−0.02	−0.13	−0.03	––												
6. US born (1 = yes, 0 = no)	0.05	−0.03	0.17	0.14	0 .14	––											
Physical functioning																	
7. Number of illnesses	−0.08	−0.01	−0.10	0 .07	0.05	0.05	––										
8. Subjective health	0.12	−0.10	−0.04	0.00	0.01	0.05	−0.19*	––									
9. PADL	−0.02	−0.15	−0.32**	−0.04	0.11	−0.06	−0.46**	−0.27**	––								
10. IADL	−0.13	−0.14	−0.11	0.06	0.08	−0.03	−0.31**	−0.17***	0.77**	––							
Cognitive functioning																	
11. MMSE	−0.18*	−0.08	−0.05	0.12	0.24**	0.12	0.15	−0.02	0.08	0.21*	––						
12. GlobDetScale^a^	−0.10	−0.03	0.06	0.00	0.03	0.09	0.18***	0.00	0.08	0.12	0.75*	––					
Social resources																	
13. Number of living children	−0.13	−0.09	0 .00	0.22*	−0.03	0.08	0.01	−0.04	−0.13	−0.03	0.03	0.04	––				
14. Relatives support	−0.11	−0.04	−0.02	0.04	0.27**	0.20***	0.01	0.11	0.04	0.08	0.10	0.07	0.27*	––			
15. Friends support	0.08	0.00	−0.01	−0.01	0.08	0.24*	0.01	0.15	0.20***	0.30**	0.14	0.07	−0.9	0.28*	––		
Mental health																	
16. GDS	0.11	0.08	−0.01	0.00	−0.04	0.02	0.20*	−0.36**	−0.37**	−0.38**	**−**0.05	**−**0.08	**−**0.15	**−**0.30*	**−**0.31*		
17. Life satisfaction	−0.01	−0.07	−0.13	0.15	−0.08	0.05	−0.11	0.26**	0.27**	0.18***	0.09	0.02	0.25*	0.20***	0.15	0.51*	––

*Residence priv* private, inst = institution. PADL = Personal Activities of Daily Living; IADL = Instrumental Activities of Daily Living; MMSE = Mini-Mental State Examination, shortened, max. 21); GlobDetScale = Global Deterioration Scale; GDS = Geriatric Depression Scale; ^a^Reverse coded for ease of interpretation; Higher values indicate better functioning (except Number of Illness and GDS)

*p < 0.01 (2-tailed); **p < 0.05 (2-tailed); ***p < 0.10 (2-tailed)

The combined regression model for depressive symptoms included the predictors subjective health, IADL, relatives support and friends support (Table 4). Friends support was not statistically significant in this combined model (*p* = .15) and was excluded. The final model explained about one third of the individual differences in depression. Subjective health was the strongest predictor, explaining 9.2 % of independent (unique) variance, followed by IADL capacity with 7.1 %, and relatives support with 4.7 %.

**Table 4:** Regression models predicting depression and life satisfaction: Domain-Specific and combined models

	Depression				Life satisfaction			
	*B*	β	p	*R* ^2^	*B*	β	p	*R* ^2^
Demographic model				.01				.05
Age	.12	.08	.43		**−**.01	**−**.03	.76	
Gender	.37	.05	.66		**−**.09	**−**.03	.74	
Residence (1 = priv, 2 = inst)	**−**.12	**−**.02	.88		**−**.38	**−**.15	.15	
Widowed (1 = yes, 0 = no)	**−**.13	**−**.02	.88		.32	.12	.21	
Education	**−**.05	**−**.03	.80		**−**.07	**−**.11	.29	
US born (1 = yes, 0 = no)	.24	.03	.76		.17	.07	.51	
Physical functioning model				.27*				.14*
Number of illness	.09	.07	.52		.04	.09	.44	
Subjective health	−1.02	**−**.33	.00		.29	.28	.01	
PADL	**−**.07	**−**.07	.64		.08	.24	.16	
IADL	**−**.20	**−**.24	.09		**−**.01	**−**.02	.90	
Cognitive functioning model				.01				.00
MMSE	.05	.04	.76		.00	**−**.01	.96	
GlobDetScale^a^	**−**.46	**−**.10	.46		.04	.03	.86	
Social resources model				.15**				.08***
Number of living children	**−**.25	**−**.10	.35		.17	.20	.07	
Relatives support score	**−**.20	**−**.20	.07		.03	.09	.43	
Friends support score	**−**.21	**−**.26	.01		.04	.14	.20	
Combined model				.28*				.20*
Subjective health	**−**.96	**−**.31	.002		.26	.25	.009	
PADL	--	--	--		.07	.22	.019	
IADL	**−**.24	**−**.27	.005		--	--	--	
Number of living children	--	--	--		.22	.24	.009	
Relatives support score	**−**.22	**−**.22	.023		--	--	--	

Variable selection for combined models was based on including all variables from domain-specific models with *p* ≤ .20. Number of predictor variables were then further reduced using a stricter criterion of .10 or less. *--* indicates that this variable has not been used in this regression model. Combined model for depression: *n* = 88. Combined model for life satisfaction: *n* = 102. ^a^Reverse coded for ease of interpretation. Higher values indicate better functioning (except Number of Illness and GDS)

*Residence priv* private, *inst* institution; *PADL* Personal Activities of Daily Living; *IADL* Instrumental Activities of Daily Living; *MMSE* Mini-Mental State Examination (shortened, max. 21); *GlobDetScale* Global Deterioration Scale; *GDS* Geriatric Depression Scale

*p < 0.01; **p < 0.05; ***p < 0.10

For life satisfaction, the combined regression model included residence type, subjective health, PADL, number of living children and friends support. Residence type (*p* =. 99) and friends support (*p* =. 38) were not statistically significant and were also excluded. The final regression model explained 20 % of the variance, which was less than the variance explained by the model for depression. The strongest predictor was subjective health explaining 5.9 % unique variance, followed by number of living children (5.7 %) and PADL (4.6 %).

## Discussion

The aim of the present study was to investigate the physical, cognitive, social and mental health of very old individuals living in New York, by determining their levels of functioning and the predictive value of demographic, cognitive and social variables for the mental health indicators depressive symptoms and life satisfaction.

Findings showed substantial health restrictions in very old age, supporting prior studies [13]. Participants had about five diagnoses, which was a slightly higher number than previously reported, suggesting a considerable illness-related burden. In contrast to their poor objective health, participants reported high subjective health, also paralleling earlier findings [33] Functional capacity was rather high for PADLs and more limited for IADLs, and both were higher than previously reported [10, 33, 34]. Male study participants did not differ in any health indicators from females, which is of note given prior work suggesting that very old men are a healthier group [6, 11, 12, 35–37]. That we found a gender difference only for marital status may be due to the rather small number of males in our sample, reflecting the gender distribution in this population.

Overall, study participants had high cognitive functioning, and only a minority demonstrated moderate impairment. This supports prior studies indicating that very old individuals are not necessarily characterized by cognitive impairment [14–16]. However, that over 90 % of the participants had few or no cognitive limitations (Global Deterioration Scale score of 1 to 3) likely reflects the fact that we included only those who were able to provide reliable self-report, which is a requirement for measuring mental health status directly from the participant. The somewhat higher cognitive capacity may also be related to the fact that the number of centenarians living in the community was substantially higher in the present study than in other US centenarian studies. In our sample, 74 % lived in private homes, whereas, for example, only 49 % lived in the community in the Georgia Centenarian Study [38]. Given that centenarians were even somewhat more likely to live in the community compared to the near-centenarians (*p* = .08), age difference between samples seems not to play a role. Alternatively, differences in residence could be related to life circumstances: As a large city, New York offers many more services, which is likely to enable the very old to live in private households for longer than in more rural communities.

In comparison to studies with younger individuals, social functioning of the near-centenarians and centenarians was poor. Although most participants had at least one person for communication/social support, their overall support score of 12 was substantially lower than that of young-olds (70 to 80 year old), who scored between 16 and 18 [29, 32]. Most participants reported two to three family members, and one to two friends within the individual support categories contact, confidant and SOS contact. Although we do not know who exactly these individuals are, it is likely that there is overlap across categories, meaning that for example the child as main go-to-person is mentioned in all categories. Half of the sample was at risk for social isolation, which was also a substantially higher proportion than in younger samples (e.g., 11 to 20 %) [29, 32]. Similar to findings from Australia centenarians [10], friends were less available than family: Compared to the younger individuals [29, 32], the very olds’ risk for social isolation was about double for the family network and about three times higher for the friend network. Unlike family members who come from different generations, friends tend to be close in terms of age; a reduced friend network is thus a likely consequence of the survivor status.

Levels of mental health were quite high, as indicated by 80 % of participants having depressive symptoms below the clinically relevant threshold and two thirds being satisfied with their lives. These findings are in line with prior centenarian studies from the US [39, 40], Australia [10, 41], and Germany [20], demonstrating considerable resilience in the very old. However, individual differences in depression and life satisfaction show that not all of them are able to maintain their mental health: That one fourth said they were only a little satisfied with their lives, and that a notable minority had depression levels suggesting a need for treatment indicates that the capacity to adapt to age-related difficulties is challenged in some very old individuals.

Our findings further added to growing work about factors related to mental health in very old age, to determine potential underlying mechanisms [19]. Significant mental health predictors came from all selected domains of functioning, which supports our comprehensive approach and recommends considering multiple domains in risk evaluation and intervention efforts. Subjective health was the strongest predictor in all regression models, whereas objective health including number of diagnoses or cognitive status did not play a role, replicating studies with younger populations [42]. Functional health was also crucial for mental health, and IADLs played a stronger role for depression, and PADLs for life satisfaction. Thus, functioning rather than number of diseases was essential for mental health. The importance of family was evident either as relatives support (depression) or number of children (life satisfaction). Differential findings make sense given that lack of support and loss of meaning are risks for depression: At age 100, family has a key role by organizing or providing care and helping with more complex activities (e.g., operating the phone), allowing for more meaningful and rewarding experiences (e.g., talking on the phone to loved ones). Given that life satisfaction is composed not only of evaluations of present aspects, but also of life achievements, it makes sense that core life accomplishments such as the number of children contribute to life satisfaction. As the very olds adaptively change their standards, being able to care for oneself at this advanced age becomes an important source of pride, reflected by the link between PADLs and life satisfaction.

One limitation of the present study is that, despite using a population-based sampling approach, only a subset of eligible centenarians participated in the study. However, the gender and racial breakdown in our study was very similar to the total US near-centenarian and centenarian population [1]. Also, since we wished to use participants’ (and not proxy respondents’) information on depression and life satisfaction, we could include only participants whose cognitive status allowed reliable self-reports. Thus, very old individuals with very poor cognitive functioning are not considered in our study. Although this has clearly resulted in a sample with a higher cognitive functioning (compared to those studies who use proxy information), we were nevertheless able to include individuals with a range of cognitive capacity by adapting our study procedures and measures to participants’ capacities. Nevertheless, the cognitive functioning in our sample is certainly higher compared to the total population of very old individuals. Furthermore, data structure does not allow for causal interpretation. Another limitation is that, due to sample size, regressions did not include all variables concurrently. Although our sequential approach resulted in reliable findings, combination of predictors may have had an influence: for example, friendship support was significant in the social model, but lost significance when including functional health (which is not surprising given the shared variance between both). Similarly, PADLs and IADLs were highly correlated, so differential predictive values have to be interpreted with care. As a final point, one needs to consider that there may be other potential aspects that could explain interindividual differences in mental health that were not included in our analysis, such as genetic factors.

## Conclusions

In conclusion, our population-based study shows that near-centenarians and centenarians are limited in many ways, most notable with respect to health and social support, yet their mental health seems to not reflect these limitations. Although study findings suggests that health evaluations, everyday competence, children and family support are among the underlying factors, future work using a similar comprehensive and multidimensional approach is needed to better understand resilience mechanisms and potential risks, ensuring that the growing number of very old experience high quality of life in their remaining years. Besides increasing our understanding, this knowledge base will furthermore help to develop support structure and services for this special age group, which take into account their specific needs, including their physical and social vulnerability as well as their preference for living in the community.

## References

[1] Meyer J (2012). Centenarians [electronic resource]: 2010 / by Julie Meyer.

[2] Christensen K, Doblhammer G, Rau R, Vaupel JW (2009). Ageing populations: the challenges ahead. The Lancet.

[3] Atzmon G, Cho M, Cawthon RM, Budagov T, Katz M, Yang X (2010). Genetic variation in human telomerase is associated with telomere length in Ashkenazi centenarians. Proceedings of the National Academy of Sciences.

[4] Ferrario A, Villa F, Malovini A, Araniti F, Puca AA (2012). The application of genetics approaches to the study of exceptional longevity in humans: Potential and limitations. Immunity & Aging.

[5] Franceschi C, Olivieri F, Marchegiani F, Cardelli M, Cavallone L, Capri M (2005). Genes involved in immune response/inflammation, IGF1/insulin pathway and response to oxidative stress play a major role in the genetics of human longevity: the lesson of centenarians. Mechanisms of Ageing and Development.

[6] Perls T (2004). Dementia-free centenarians. Experimental Gerontology.

[7] Sebastiani P, Solovieff N, DeWan AT, Walsh KM, Puca A, Hartley SW (2012). Genetic signatures of exceptional longevity in humans. PLoS ONE.

[8] Terry D, Sebastiani P, Andersen S, Perls T (2008). Disentangling the roles of disability and morbidity in survival to exceptional old age. Arch Inter Med.

[9] Willcox D, Willcox B, Hsueh W, Suzuki M (2006). Genetic determinants of exceptional human longevity: insights from the Okinawa Centenarian Study. Age (Dordr).

[10] Richmond RL, Law J, Kay-Lambkin F (2011). Physical, mental, and cognitive function in a convenience sample of centenarians in Australia. Journal of the American Geriatrics Society.

[11] Evert J, Lawler E, Bogan H, Perls T (2003). Morbidity profiles of centenarians: survivors, delayers, and escapers. Journals of Gerontology Series A: Biological Sciences & Medical Sciences.

[12] Andersen SL, Sebastiani P, Dworkis DA, Feldman L, Perls TT (2012). Health span approximates life span among many supercentenarians: Compression of morbidity at the approximate limit of life span. Journals of Gerontology - Series A Biological Sciences and Medical Sciences.

[13] Andersen-Ranberg K, Schroll M, Jeune B (2001). Healthy centenarians do not exist, but autonomous centenarians do: a population-based study of morbidity among Danish centenarians. Journal of the American Geriatrics Society.

[14] Gondo Y, Poon LW (2007). Cognitive function of centenarians and its influence on longevity. Annual Review of Gerontology & Geriatrics.

[15] Silver MH, Jilinskaia E, Perls TT (2001). Cognitive functional status of age-confirmed centenarians in a population-based study. Journals of Gerontology - Series B Psychological Sciences and Social Sciences.

[16] Kliegel M, Moor C, Rott C (2004). Cognitive status and development in the oldest old: A longitudinal analysis from the Heidelberg Centenarian Study. Archives of Gerontology and Geriatrics.

[17] Martin P, Poon LW, Clayton GM, Lee HS, Fulks JS, Johnson MA (1992). Personality, life events and coping in the oldest-old. International Journal of Aging and Human Development.

[18] Randall GK, Martin P, McDonald M, Poon LW (2010). Social resources and longevity: Findings from the Georgia centenarian study. Gerontology.

[19] Poon LW, Miller LS, Martin P, Cho J, Da Rosa G, Deshpande N, Margrett J, Bishop A, Hensley R, MacDonald M et al. Understanding centenarians' psychosocial dynamics and their contributions to health and quality of life. Current Gerontology and Geriatrics Research 2010, 2010. http://dx.doi.org/10.1155/2010/680657 10.1155/2010/680657 PMC2948878 20936141

[20] Jopp D, Rott C (2006). Adaptation in very old age: Exploring the role of resources, beliefs, and attitudes for centenarians' happiness. Psychology and Aging.

[21] Ravaglia G, Forti P, Maioli F, Boschi F, Cicognani A, Bernardi M (1997). Determinants of functional status in healthy Italian nonagenarians and centenarians: A comprehensive functional assessment by the instruments of geriatric practice. Journal of the American Geriatrics Society.

[22] Martin P, Rott C, Kerns MD, Poon LW, Johnson MA (2000). Predictors of depressive symptoms in centenarians. Facts, Research and Intervention in Geriatrics.

[23] Wong WC, Lau HP, Kwok CF, Leung YM, Chan MY, Chan WM (2014). The well-being of community-dwelling near-centenarians and centenarians in Hong Kong: a qualitative study. BMC geriatrics.

[24] Jopp DS, Liu Y, Wozniak D, Rott C, Lehrfeld J. Personal resources and meaning in life predict depressive symptoms: findings from the Heidelberg Centenarian Study. University of Lausanne. 2014.

[25] Fillenbaum GG (1988). Multidimensional functional assessment of older adults: The Duke Older Americans Resources and Services procedures.

[26] Folstein MF, Folstein SE, McHugh PR (1975). 'Mini mental state'. A practical method for grading the cognitive state of patients for the clinician. Journal of Psychiatric Research.

[27] Holtsberg PA, Poon LW, Noble CA, Martin P (1995). Mini-Mental State Exam status of community-dwelling cognitively intact centenarians. International Psychogeriatrics.

[28] Reisberg B, Ferris SH, De Leon MJ, Crook T (1982). The global deterioration scale for assessment of primary degenerative dementia. American Journal of Psychiatry.

[29] Lubben JE (1988). Assessing social networks among elderly populations. Family & Community Health: The Journal of Health Promotion & Maintenance.

[30] Sheikh JI, Yesavage JA (1986). Geriatric Depression Scale (GDS): Recent evidence and development of a shorter version. Clinical Gerontologist.

[31] Pavot W, Diener E (1993). Review of the Satisfaction With Life Scale. Psychological Assessment.

[32] Lubben J, Blozik E, Gillmann G, Iliffe S, von Renteln KW, Beck JC (2006). Performance of an abbreviated version of the Lubben Social Network Scale among three European community-dwelling older adult populations. The Gerontologist.

[33] Schönemann-Gieck P, Rott C, Martin M, D'Heureuse V, Kliegel M, Becker G (2003). Similarities and differences between self-rated and proxy-rated health in extreme old age. Übereinstimmungen und unterschiede in der selbst- und fremdeingeschätzten gesundheit bei extrem hochaltrigen.

[34] Motta M, Ferlito L, Magnolfi SU, Petruzzi E, Pinzani P, Malentacchi F (2008). Cognitive and functional status in the extreme longevity. Archives of Gerontology and Geriatrics.

[35] Samuelsson SM, Alfredson BB, Hagberg B, Samuelsson G, Nordbeck B, Brun A (1997). The Swedish Centenarian Study: a multidisciplinary study of five consecutive cohorts at the age of 100. International journal of aging & human development.

[36] Perls TT, Silver MH, Lauerman JF (1999). Living to 100: Lessons in living to your maximum potential at any age.

[37] Tigani X, Artemiadis AK, Alexopoulos EC, Chrousos GP, Darviri C (2011). Gender differences in Greek centenarians. A cross-sectional nation-wide study, examining multiple socio-demographic and personality factors and health locus of control. BMC geriatrics.

[38] Margrett J, Martin P, Woodard JL, Miller LS, MacDonald M, Baenziger J (2010). Depression among centenarians and the oldest old: Contributions of cognition and personality. Gerontology.

[39] Adkins G, Martin P, Poon LW (1996). Personality traits and states as predictors of subjective well-being in centenarians, octogenarians, and sexagenarians. Psychol Aging.

[40] Bishop AJ, Martin P, MacDonald M, Poon L (2010). Predicting happiness among centenarians. Gerontology.

[41] Sachdev PS, Levitan C, Crawford J, Sidhu M, Slavin M, Richmond R (2013). The Sydney Centenarian Study: methodology and profile of centenarians and near-centenarians. International Psychogeriatrics.

[42] Smith J, Fleeson W, Geiselmann B, Settersten RA, Kunzmann U, Baltes PB, Mayer KU (1999). Sources of wellbeing in very old age. The Berlin Aging Study: Aging from 70 to 100.

